# Prevalence of Polypharmacy Among Patients with Chronic Liver Disease—A Narrative Literature Review

**DOI:** 10.3390/jcm14176263

**Published:** 2025-09-05

**Authors:** Monika Szkultecka-Dębek, Lucyna Bułaś, Agnieszka Skowron, Mariola Drozd

**Affiliations:** 1 University of Social Sciences, 00-842 Warsaw, Poland; mszkultecka-debek@san.edu.pl; 2 Department of Pharmaceutical Technology, Medical University of Silesia in Katowice, 41-200 Sosnowiec, Poland; lbulas@sum.edu.pl; 3 Department of Social Pharmacy, Jagiellonian University Medical College, 30-688 Krakow, Poland; agnieszka.skowron@uj.edu.pl; 4 Department of Humanities and Social Medicine, Medical University of Lublin, 20-093 Lublin, Poland

**Keywords:** polypharmacy, liver disease, drug-induced liver injury, adverse outcomes, medication-related problems

## Abstract

**Background and aim:** Managing the therapy of patients with chronic liver diseases and comorbidities presents significant challenges for physicians and pharmacists, particularly regarding drug-induced liver damage and polypharmacy. Given the liver’s central role in drug detoxification, polypharmacy in liver disease requires special attention. The aim of the review was to assess the prevalence of polypharmacy among patients with chronic liver diseases. **Approach and Results:** A literature search focused on randomized controlled trials, database reviews, and medical records. Review of PubMed, SCOPUS, and ScienceDirect databases identified 2578 manuscripts, however only 11 studies met the inclusion criteria. The results of studies showed that the prevalence of polypharmacy among patients with chronic liver disease can exceed 50%, and can lead to high prevalence of MRP and pDDI among those patients. **Conclusions:** Findings reveal a critical link between polypharmacy and adverse outcomes in chronic liver diseases, including cirrhosis, hepatitis, and non-alcoholic fatty liver disease. Individualized treatment plans, considering factors such as age, gender, comorbidities, and liver disease severity are essential. The interventions focused on mitigating MRP and reducing pDDI need to be implemented in order to reduce the potential harm of polypharmacy.

## 1. Impact and Implications

Maintaining patient safety during pharmacotherapy is the responsibility of physicians and pharmacists. The use of polypharmacotherapy presents a particular challenge in the treatment of chronic liver disease—an organ that is the primary site of drug metabolism. Effective interventions by doctors and pharmacist hold promise for reducing MRPs and mitigating risks associated with pDDIs. This review reveals a critical link between polypharmacy and adverse outcomes in chronic liver diseases. Although polypharmacy is a recognized concern, with an increasing ongoing discussion it remains essential to raise awareness and drive effective solutions. This publication focuses on improving pharmaceutical knowledge related to polypharmacy and its safety.

## 2. Introduction

Managing treatment of patients with chronic diseases coexisting with chronic liver damage and liver diseases poses a challenge for physicians and pharmacists. It is known that pharmacotherapy may potentially induce liver injuries. As one of the US studies show drug induced liver injury (DILI) can be responsible for as many as 50% of acute liver failures [1]. Despite emerging scientific reports on the diagnosis of clinical conditions such as DILI and non-alcoholic fatty liver disease (NAFLD), these diseases still constitute a real problem, in particular when using drugs in the absence of a prior diagnosis of liver dysfunction.

NAFLD, being associated with obesity and with the diagnosed metabolic syndrome, may also affect the drugs clearance and it has similar pathophysiological mechanisms as the drug-induced liver injury, which may lead to drug-induced hepatotoxicity [2,3].

Moreover, it is worth to mention the existence of many liver diseases’ microbiological basis. This should be reflected in the development of diagnostic tests related to the condition of the liver and also in relation to the intestinal microbiota [4]. It is important to note that NAFLD is diagnosed as standard procedure by imaging, usually by performing an abdominal ultrasonography (USG) [5]. However, also in this case it is necessary to exclude the secondary fatty liver disease. Despite indicating the aminotransferases activity (ALT, AST) and their increased level, it has been proven that in 60% of the examined cases these tests are not reliable since the tested parameters may remain within normal limits, even in patients with NAFLD. Therefore, it is assumed that ALT activity is not a biomarker of NAFLD or non-alcoholic steatohepatitis (NASH) and has no diagnostic significance in NASH or liver fibrosis. It is worth emphasizing that normal ALT activity does not exclude NAFLD and cannot be the basis for its diagnosis [6,7].

It is also worth noting that the COVID-19 pandemic has exposed the threat of polypharmacy, causing multiple hepatotoxic effects, as confirmed by Badary et al. [8]. Some of the scientists in their reports emphasize the importance of performing polypharmacy audits in order to recognize and prevent potential drug-related problems (DRPs) in specific patients’ groups, e.g., those with diagnosed liver cirrhosis. This problem becomes particularly important in elderly patients, where, according to the authors of the report on the criteria for the correctness of geriatric pharmacotherapy in clinical practice, in order to optimize geriatric pharmacotherapy, it is necessary to regularly review all medications and dietary supplements taken by elderly patients [9]. The implementation of strategies to optimize the use of medicines in patients, also those with liver cirrhosis, has been repeatedly pointed out in different studies, emphasizing that these activities have a significant impact on both, the length of patients’ hospital stay and morbidity [6].

The liver diseases may also be a consequence of unfavourable drug interactions that change the effectiveness of the therapy used, increasing the risk of drug-related diseases affecting patients’ health. Despite the existence and easy access to the online programs dedicated to drug interactions some authors point out that those tools do not take into account all the factors, such as the currently used dose, and therefore they do not prevent a number of potential interactions. Emerging difficulties in the treatment of patients with multimorbidity require taking the patient’s condition into account. According to different authors, the unfavourable drug combinations limit their effectiveness and may induce the appearance of new symptoms, which are often interpreted as a new disease, which leads to initiation of a new treatment [10].

Currently, in Poland, there are over 22,000 medicinal products approved for trading. In 2023, 576 medicinal products were authorized by the national procedures [11]. For most of those products liver is the main detoxification organ. According to literature data approximately 1000 different drugs and other substances with potential hepatotoxic effects have been identified. During a study conducted in the United States there were 671 drugs analyzed, and within that group 53% of products showed liver-damaging effects [12]. Drugs that potentially damage the liver act through the direct hepatotoxic effect, depending on the dose of the drug taken and a specific latency period, usually 1–8 weeks. According to Woroń et al., diseases occurring in patients modify the pharmacokinetic and pharmacodynamic profile of the drugs used. Typical examples of such diseases include liver disease, kidney disease and type II diabetes [13].

Patient-dependent factors of DILI include: age (the older the patient, the higher risk of hepatotoxicity, which is related to the lower activity of liver metabolism), gender (women are more likely to suffer from the disease), pregnancy, ethnicity, genetic factors, the presence of comorbidities, especially chronic liver diseases or the metabolic syndrome [2,14,15]. The drug-dependent risk factors include: dose (single, daily, cumulative dose in some drugs), chemical structure, pharmacokinetic profile (lipophilicity) and hepatic metabolism, effect on mitochondrial function and hepatobiliary transport, concomitant use of other drugs and the risk interactions (especially in the case of drugs metabolized by cytochrome P-450 isoenzymes) [16,17].

In a retrospective cohort study conducted in the medical departments of two tertiary hospitals in Pakistan, Sidra Noor and co-authors observed 413 patients with hepatitis presenting with a significant number of clinically significant pDDI (potential drug-drug interaction). The study was conducted for screening purposes using specific pDDI criteria with the level of importance defined as: contraindicated, serious, moderate and minor. The study referred to the process of documenting therapy as an important element in communication when transferring a patient to treatment by medical teams. Authors as conclusion pointed out to the widespread use of major pDDI drugs and their potential adverse effects. According to the findings, clinically important parameters, such as laboratory results and signs or symptoms, should be monitored, especially in case of high-risk patients treated with polypharmacy, when the patient experienced a prolonged hospitalization or had a stroke as a comorbid condition [18]. Therefore, in pharmaceutical and physician practice, it is extremely important to thoroughly analyze the medicinal products taken by the patient and carefully consider the indications and contraindications to the use of the product, especially in the case of diagnosed liver pathologies. As in the case of many diseases, also in relation to the acute and chronic hepatic diseases the basis for health improvement can be prevention of polypharmacy. The aim of the review was to assess if the polypharmacy exists among patients with diagnosed chronic liver damage and diseases.

## 3. Materials and Methods

For the purpose of this work, a review of the literature and published studies related to the use of polypharmacy in comorbid liver diseases was performed. The attempts were made to apply the methodology of conducting a systematic review, however, the first stage of the initial search of the databases was performed by one researcher, while the assessment and selection of identified articles was carried out by two independent researchers. The results are reported in accordance with the Preferred Reporting Items for Systematic Review and Meta-analysis (PRISMA) guidelines [19].

**Figure 1:**
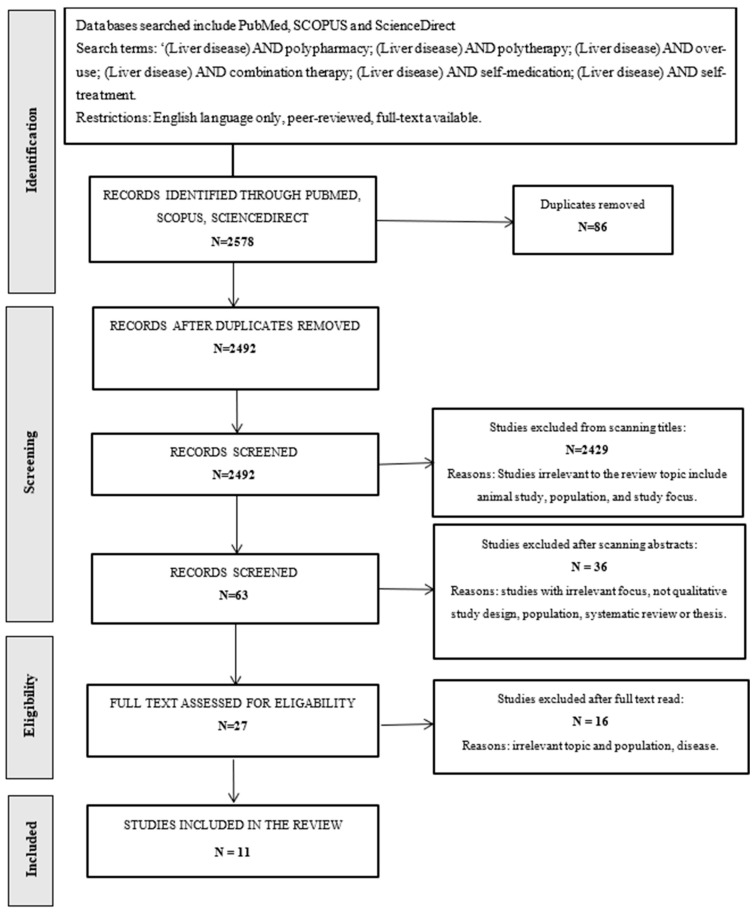
PRISMA Flowchart for study search and selection for literature review.

The search strategy was run on three data-bases (PubMed: 1990 to 2024, SCPOUS: 1977 to 2024 and Science Direct: 1999 to 2024) and according to the following search criteria: (Liver disease) AND polypharmacy; (Liver disease) AND polytherapy; (Liver disease) AND overuse; (Liver disease) AND combination therapy; (Liver disease) AND self-medication; (Liver disease) AND self-treatment. Database initial searches were performed between 20–25 August 2022 and updated on 10 October 2024. The inclusion criteria were limited to primary research studies, including adult patients with an existing diagnosis of a chronic liver disease and the use of polypharmacy. Specifically the focus was on randomized controlled trials (RCTs), database reviews and medical records reviews. The systematic reviews were excluded in order to avoid duplication bias, as they synthesize primary studies and may lead to overrepresentation of the findings. Additionally, studies involving animals, populations not relevant to the target group and case reports were excluded from the review due to limited generalizability and methodological constraints. This strategy presented in Figure 1.

## 4. Results

Based on the search there were 2578 published manuscripts identified, however after removing the duplicated studies, the irrelevant studies which had different focus, were performed in different populations or on animals, those not fulfilling the inclusion criteria, and the manuscripts dedicated to case studies or systematic reviews the final number of studies included in the review were eleven. The details of the study results are presented in Table 1.

The results show that the prevalence of polypharmacy among patients with chronic liver disease is high and does not depend on type of chronic liver damage or disease. The prevalence of polypharmacy was higher than 50% in patients with cirrhosis and NAFLD as it was shown in some studies [20,22,25,26,28]. The Hudson et al. publication showed that patients with chronic hepatitis C have higher level of polypharmacy and according to Patel et al. 90% of patients with NAFLD took regularly 5 or more medications. The studies also revealed high prevalence of MRP or DRP, and among them high rate of pDDI [18,22,23,27,29]. Polypharmacy was also correlated with lower quality of life level [26,30].

## 5. Discussion

Since liver is a metabolically active organ, the analysis of reports on studies investigating its function across various research centers is important, especially with a focus on risk factors for liver damage. The results of the performed review show a high prevalence of polypharmacy among patients with chronic liver disease independently of the type of chronic liver damage or disease. The review also found that polypharmacy negatively impacts the level of patients’ quality of life.

In this context it is worth noticing that among the well-known and frequently cited causes of liver dysfunction are medications, particularly their metabolites, as well as other agents used in patient therapies, such as dietary supplements, herbal medicines, and patients’ dietary habits. Despite the accumulated data and the existing knowledge, in clinical practice serious liver damage still continues to occur and is observed in relation to prescribed treatments.

To address the issue of polypharmacy in patients’ care, it is essential to consider individual predispositions such as advanced age, gender, pregnancy, severe malnutrition, metabolic diseases (e.g., diabetes, obesity), and infections like HBV, HCV, or HIV. Additional factors such as smoking, stress, and environmental pollution can further alter the liver’s metabolic profile. Based on the published data it can be concluded that the drug-induced liver injuries are more frequently observed in females [30]. Liver injuries may involve hepatocytes and mitochondria through various pathways, including immune responses.

Moreover, it is worth noting that the importance of influence and impact on the management of polypharmacy has been underestimated. A cross-sectional study conducted remotely with physicians and hospital pharmacists in 20 provinces of China showed that both groups have knowledge, positive attitudes, and proactive practices towards polypharmacy in the elderly with chronic diseases. The results of this study emphasize the need for education, collaboration among professionals, and a regular assessment of the quality of care for elderly patients with chronic diseases [31].

Yu K. et al. in their research state that medicinal products that are substrates of CYP450 enzymes are more likely to develop drug-induced liver damage, regardless of the dose. However, in relation to inhibitors of this enzyme, the likelihood of liver damage associated with high doses of drugs is suggested [32]. It is also known that the CYP 3A4 isoform is responsible for the metabolism of over 50% of all drugs and it should be emphasized that CYP450 expression depends, among other factors, on genetic polymorphisms. In case that a given isoenzyme is inactive, those patients are classified as slow drug metabolizers. The expression of CYP450 isoenzymes also depends on gender, where men have a faster metabolism, and on the age of the patients, demonstrating lower activity of liver microsomal enzymes in newborns and among geriatric patients [33].

Despite many studies related to drugs hepatotoxicity and liver damage, to this day there is no single, characteristic marker allowing for optimal diagnosis of drug-induced liver injuries. In clinical practice the diagnostic procedure mainly involves testing the concentration of total and conjugated serum bilirubin, assessing ALP (alkaline phosphatase), ALT (alanine transaminase) and AST (aspartate aminotransferase) activity and concentration. However, the described markers are not specific for drug-induced liver damage. Thus, an increase in the activity of the ALT enzyme can be observed in a number of diseases, such as: cancer, viral hepatitis, alcoholic fatty liver disease, heart muscle damage or excessive exercise and an increased bilirubin levels also occur in thyroid and bone diseases [34]. 

In relation to this, it is worth mentioning the work of Tveden-Nyborg et al., who discuss the lack of specific biomarkers and the need for better diagnostic tools. According to the literature review performed by Tveden-Nyborg research into signalling pathways involved in DILI is needed in order to improve both, the biomarkers and the treatment options for DILI patients. The authors also point out the importance of including patients with DILI-induced by dietary supplements and herbal medicines [1].

The gold standard for predicting drug-induced liver damage in clinical practice is defined by Hy’s law, consisting on a comparison of ALT activity (three times above normal), serum bilirubin concentration (twice above normal) with a concurrent jaundice. According to Zimmerman H., patients suffering from DILI-induced icterus have at least a 10% risk of developing acute liver failure, regardless of which drug caused the organ damage. However, a modification of these assumptions is required, which may be facilitated by new biomarkers of drug-induced liver injury, contributing to improve the effectiveness of DILI detection [35].

The incidence of drug-induced liver damage is estimated at 1 in 1000 patients [36]. However, as mentioned, despite numerous studies, the pathogenesis of liver damage remains incompletely understood and needs more research and attention.

The identified data during the performed literature search highlights the critical relationship between polypharmacy and adverse outcomes in patients with chronic liver diseases, including cirrhosis, hepatitis, and NAFLD. Polypharmacy is prevalent across the studied populations, with utilization rates ranging from 56.3% to over 90%, depending on the patient group. The frequent use of multiple medications is associated with significant risks, including adverse drug reactions (ADRs), potential drug-drug interactions (pDDIs), and a decline in quality of life (QoL).

Several studies have focused on the prevalence of polypharmacy and its association with adverse outcomes, e.g., authors of a systematic review and meta-analysis reported a pooled polypharmacy prevalence of 37% among individuals over age 19, with higher rates observed in older adults and inpatient settings [37]. Another review, focused on polypharmacy, found that its prevalence varied between 10% to as high as around 90% in different populations, influenced by factors such as chronic conditions, demographics, and socioeconomics, concluding that optimising care for polypharmacy will improve the health outcomes in older patients [38]. Despite that those reviews were not focused on specific liver diseases, as mere research, they provide important input into the polypharmacy impact on health outcomes.

Based on the review that we performed it is worth to mention that patients with polypharmacy experience a disproportionately higher incidence of complications. For example, in a study within a group of cirrhotic patients, 56.3% of them used polypharmacy, nearly 36% were requiring treatment monitoring and 3.3% were prescribed with drugs that should be avoided. Older patients and those with greater comorbidity burdens, including renal dysfunction and hepatic decompensation, showed higher adjusted odds ratios (RORs) for adverse events and medication-related problems (MRPs). Based on the data, patients with ADRs are usually older, having more comorbidities, are treated with more drugs and have worse renal function [21].

Also, pDDIs were frequently reported, particularly in patients prescribed with more than nine medications, with significant associations to extended hospitalizations and comorbid conditions such as stroke. For hepatitis patients, 55.2% experienced pDDIs, and among chronic hepatitis C patients, psychotropic agents, antidiabetics, and statins were the most commonly used. The presence of pDDIs not only exacerbated liver dysfunction but also contributed to hospitalizations and worsened the clinical outcomes [18].

It is worth mentioning that in NAFLD patients, polypharmacy correlated with significantly reduced physical and mental health scores. This pattern was also evident in patients with advanced liver disease, where polypharmacy negatively affected QoL and further compromised health outcomes. E.g. a study conducted in the USA showed that NAFLD patients taking five or more medications had significantly more symptoms and lower quality of life, compared to the non-polypharmacy group, which was confirmed by Alrasheed et al. In terms of the quality of life domains, the negative impact on physical health was mainly due to symptoms such as fatigue and muscle weakness, while the negative impact on mental health was mainly due to depression/sadness and fatigue [39].

In relation to the pharmacist role, according to Zaij et al., the multidisciplinary drug review meetings, including at least the physician/pharmacist/nurse trio, is the most common multidisciplinary intervention which may prevent adverse drug events in the adult population [40].

Hayward and Weersink emphasize the need of regular reviews of the prescribed therapies. That is essential to reduce the unnecessary drug burden and enable for identification of the medicinal products which might be used inappropriately in the specific case [41].

In our final evaluation, selected studies presented in Table 1, are listed by factors comparing liver results, which include: cirrhosis, inflammation, and non-alcoholic fatty liver disease. Treatment, including common comorbidities, is challenging. Patients often struggle with difficulties related to treatment regimens and the need to verify prescribed medications by different physicians who usually do not have access to full patients medical data. In justifying the guidelines for implementing post-hospital pharmaceutical care into pharmaceutical practice, the authors pointed to errors identified by clinical pharmacists, including: missed medication, incorrect dose, incorrect dosing regimen, formulation, duration of therapy, incorrect medication, polypharmacy, and lack of information on how to use the medication [42,43,44].

Pharmaceutical consultations New Medical Service (NMS) conducted in many European countries demonstrate that NMS can in an optimal and comprehensive way influence patients’ health behaviors, increasing the adherence to treatment recommendations. NMS increases patients’ knowledge and skills in the field of new drug therapy, while allowing pharmacists to independently make changes or suggest modifications to their pharmacotherapy to ensure it is as convenient and safe as possible for the patient [45].

Another activity of a pharmacist within the area of pharmacovigilance is conducting a Minor Ailments (MA) pharmaceutical consultation. Aiming to select medicinal products for a patient visiting the pharmacy due to the occurrence of symptoms that do not require medical consultation or an in-depth diagnosis. The World Health Organization (WHO) indicates that providing assistance in the event of minor ailments is an important element of primary health care in the area of self-care and independent use of medications by patients. The responsibility of the pharmacist when dispensing over-the-counter medications, especially to patients with liver disease, is to provide comprehensive information that will enable the patient to use these products in a proper and safe way [46].

In many countries around the world Medication Reviews are performed. Due to the varying operating conditions of healthcare systems and the pharmacist’s position within them, those reviews are conducted in the outpatient and inpatient settings, and can be performed not only in a pharmacy, clinic, or hospital but also at the patient’s place of residence. The pharmacist conducting medication reviews enables the detection of potential and actual medication problems related to the current pharmacotherapy, particularly in patients treated with polypharmacy. The pharmacist develops preventative measures or solutions to medication problems that will optimize pharmacotherapy and increase the patient’s chances of achieving the best possible therapeutic outcomes [47].

In the context of the discussed issue, it is worth noting that pharmacists’ interventions significantly contributed to shortening the duration of re-hospitalization, improving adherence and knowledge about the medications used [48,49,50,51,52,53,54,55,56].

A proper pharmacist’s support at every stage of the therapy can help to improve the adherence to medical recommendations, reduce the risk of re-hospitalization and increase the effectiveness of ordered treatment [57].

Among the published data among others, it is worth to look into Farook et al. research dedicated to polypharmacy, where the authors recommend as the ideal option an individualized approach to treatment. The presented suggestion is to use clinical pharmacy service and regularly review medication regimens in order to optimize the treatment. Such approach will lead to the elimination of drugs duplication and reduction of the dosing frequency [29].

## 6. Conclusions

Polypharmacy is an inherent challenge in the management of chronic liver diseases, with substantial implications for patient safety and clinical outcomes. The evidence underscores the necessity of individualized treatment plans that incorporate risk stratification based on patient-specific factors such as age, gender, comorbidity burden, and liver disease severity.

Effective interventions, including pharmacist involvement, hold promise for reducing MRPs and mitigating risks associated with pDDIs. Future efforts should focus on optimizing prescribing practices, enhancing monitoring strategies, and implementing multidisciplinary approaches to minimize the burden of polypharmacy while maintaining therapeutic efficacy. Additionally, an increased awareness of the adverse impacts of polypharmacy on patients’ QoL is required.

Recommendations for clinical practice and Policy:

Structured, standardized medication review protocols should be implemented into clinical practice as a routine procedure in case of chronic liver disease management. Pharmacist-led services integrated into hepatology clinics providing oversight of the complex treatment regimens would be beneficial to both, patients and clinicians. Health policies should support the multidisciplinary approach.

## Figures and Tables

**Table 1:** Characteristics and results description of the studies included in the review.

Authors/Title	Objective/Evidence Type	Population/Results
Elzouki et al.: Polypharmacy and drug interactions amongst cirrhotic patients discharged from a tertiary center: Results from a national quality improvement audit. (2020) [20]	Audit the drug prescribed in patients with cirrhosis and analyze the quantity and severity of potential drug-drug interaction/Electronic Medical Records review	Adult patients, diagnosed with cirrhosis. Relevant data from 181 patients. Average drug utilization 7.8 ± 3.1 (range = 1–17). 102 (56.3%) patients used polypharmacy. The drug should be avoided in case of 3.3%, 16.6% of cases to consider therapy modification, 35.9% to monitor treatment. Utilization of polypharmacy was statistically significant in patients where drug should be avoided (83.3%, *p* = 0.03).
Abe et al.: Analysis of polypharmacy effects in older patients using Japanese adverse drug event report database. (2017) [21]	Examined AE profiles associated with polypharmacy and aging; association between polypharmacy and “renal disorder” or “hepatic disorder”/Database review	Older patients, hepatic, disorder. For hepatic disorder, the adjusted RORs (reported odds ratio) were as follows: 1.17 (1.14–1.20) for the number of administered drugs group (5–9) and 1.14 (1.11–1.18) for the number of administered drugs group (≥10). The adjusted RORs of hepatic disorder compared to those of renal disorder had lower adjusted RORs related to the increase in the number of administered drugs.
Franz et al.: Potential drug-drug interactions and adverse drug reactions in patients with liver cirrhosis. (2012) [22]	Assess risk for potential drug-drug interactions (pDDIs) and/or adverse drug reactions (ADRs) due to the severity of disease and comorbidities associated with polypharmacy/Electronic Medical Records review	Adult patients with cirrhosis. There were 400 patients with cirrhosis, 6 (1–10) diagnoses per patient; 60.7% of the diagnoses were not liver-associated; median number of drugs per patient: 5 (0–18), whereof 3 (0–16) were predominantly hepatically eliminated. Prescribed drugs were primarily indicated for gastrointestinal, cardiovascular, or nervous system disorders, reflecting the prevalent diagnoses. 28% patients had 200 ADRs; 21.5% patients had 132 pDDIs. 7 pDDIs were cause of 15 ADRs and 3 resulted in hospitalization. Patients with ADRs: older, more comorbidities, treated with more drugs, worse renal function, more pDDIs.
Hayward et al.: Medication-Related Problems in Outpatients With Decompensated Cirrhosis: Opportunities for Harm Prevention. (2019) [23]	Assessed the association between MRPs (medication related problems) and patient outcomes/Randomized clinical trial	Patients with a history of decompensated cirrhosis. There were 57 intervention patients; 375 MRPs identified; most prevalent MRP types: nonadherence (31.5%) and indication issues (29.1%); risk of potential harm associated with MRPs was low in 18.9% of instances, medium in 33.1%, and high in 48.0%, as categorized by a clinician panel using a risk matrix tool. Patients had a greater incidence rate of high-risk MRPs if they had a higher Child-Pugh score (incidence rate ratio [IRR], 1.31; 95% confidence interval [CI], 1.09–1.56); greater comorbidity burden (IRR, 1.15; 95% CI, 1.02–1.29); and were taking more medications (IRR, 1.12; 95% CI, 1.04–1.22). Pharmacist intervention resulted in the resolution of 58.9% of MRPs.
Noor et al.: Drug-drug interactions in hepatitis patients: Do these interactions matter in clinical perspectives? (2018) [18]	Explored frequency, levels, predictors, and clinical relevance of pDDIs (potential drug-drug interactions) in hospitalized hepatitis patients./Retrospective cohort study	Hepatitis patients. A total of 413 hepatitis patients, 55.2% reported pDDIs; total of 660 pDDIs; 35% patients—major pDDIs. Significant association for the presence of pDDIs with > 9 prescribed medicines (*p* < 0.001), hospitalization of >5 days (*p* = 0.03), and stroke as comorbidity (*p* = 0.05). Significantly higher odds of exposure to major-pDDIs in patients taking > 9 prescribed medicines (*p* < 0.001), hospitalization of > 5 days (*p* = 0.002), and stroke as comorbidity (*p* = 0.002).
Ruzicka et al.: Comorbidities and co-medications in populations with and without chronic hepatitis C virus infection in Japan between 2015 and 2016. (2018) [24]	To examine number and types of comorbidities and co-medications by age group in patients with and without chronic HCV/Retrospective, observational hospital database study	Patients with chronic HCV and non-HCV patients. There were 128,967 chronic HCV patients and 515,868 non-HCV patients. The most common comorbidities in chronic HCV patients were diseases of oesophagus, stomach and duodenum (41.7%), followed by hypertensive diseases (31.4%). Chronic HCV patients and older patients used more co-medications. 41.9% of chronic HCV patients and 26.0% of non-HCV patients used at least one co-medication supplied for ≥180 days or recorded in at least 6 consecutive months. Among chronic HCV patients, 19.0% used 1–3 co-medications, 11.0% used 4–6, and 11.9% used ≥7 co-medications, compared with 13.8, 6.5, and 5.8% of non-HCV patients. 16.2% of chronic HCV patients aged 80–84 years used ≥7 co-medications. The most common long-term co-medications in chronic HCV patients were proton pump inhibitors (prescribed to 31.9% of chronic HCV patients at least once during the study period).
Alrasheed et al.: The effect of polypharmacy on quality of life in adult patients with nonalcoholic fatty liver disease in the United States. (2022) [25]	To examine the association between polypharmacy and health-related quality of life (QoL) in NAFLD adult patients/Retrospective observational study	Nonalcoholic fatty liver disease (NAFLD) adult patients. A total of 1067 NAFLD adult patients; 834 patients with polypharmacy; patients with NAFLD and polypharmacy have lower QoL than those with NAFLD and nonpolypharmacy. Number of medications had a significant negative impact on PCS (physical component summary), MCS (mental component summary), and all SF-36 domains except mental health, role physical limitation and role emotional limitation domains.
Patel et al.: Multimorbidity and polypharmacy in diabetic patients with NAFLD. Implications for disease severity and management. (2017) [26]	Identifying characteristics that may impact liver disease severity or clinical management of patients with diabetes and NAFLD/Observational study	Adult patients with diabetes 2 and with NAFLD. A total of 95 patients; 10% took <5 regular medications; 59% polypharmacy (5–9 medications); 31% hyperpolypharmacy (≥10 medications). Older patients and patients with a history of IHD or osteoarthritis were taking more medicines (*p* = 0.01, *p* <0.01, and *p* = 0.05, respectively). Significant relationship between number of medications taken and number of co-morbidities.
Hudson et al.: Comorbidities and medications of patients with chronic hepatitis C under specialist care in the UK. (2017) [27]	Using patient data to describe the demographics currently under specialist hepatology care who are likely to be eligible for direct-acting antiviral (DAA) treatment over the next 5 years, and investigate the prevalence of comorbidities, adverse lifestyle factors, and use of medications with potential DDIs/Retrospective analysis. Data from National HCV Research UK Biobank	Adult chronic hepatitis C. A total of 6278 patients (70.5% white; median age, 52 years) from 59 UK specialist centres were included; 59.1% of patients had acquired HCV through injecting drug use (IDU). The most common medications with drug-drug interaction (DDI) potential were psychotropic agents (antidepressants, opioids, and hypnotics) (38.6%), antidiabetics (9.3%), immunosuppressants (6.1%), statins (4.9%), and antiretrovirals (4.9%). This study concurs that patients with CHC in the UK have high levels of non-HCV comorbidity and polypharmacy.
Hayward et al.: Changing Prevalence of Medication Use in People with Cirrhosis: A Retrospective Cohort Study Using Pharmaceutical Benefits Scheme Data. (2023) [28]	To characterise the prescriptions dispensed to people with cirrhosis and explore changes in the use of medication groups over time./Observational study/Data from a multi-site, prospective, observational study	Patients with diagnosed cirrhosis; 522 patients (mean age 60 years, 70% male, 34% decompensated at recruitment), 89,615 prescriptions during the follow-up period; median of 136 prescriptions and a median of 16 unique medicines per patient (total =9306 medicines). The most commonly used medicines were proton pump inhibitors (dispensed at least once to 73% of patients), opioids (68%) and antibiotics (89%). Polypharmacy: 59–69% of participants in each time period dispensed five or more unique medicines. Prescription medication use increased over time (*p* < 0.001) independently of age, comorbidity burden and liver disease aetiology.
Farooq et al.: Polypharmacy in chronic liver disease patients: Implications for disease severity, drug-drug interaction, and quality of life. (2023) [29]	To evaluate polypharmacy in patients with chronic liver disease and to identify potential drug-drug interactions associated with it/Cross-sectional study	Patients with chronic liver disease from various age groups. Number of prescribed drugs significantly correlated (*p* = 0.018) with the severity of liver disease in Child-Pugh categories B and C. Moderate polypharmacy reduced quality of life (*p* < 0.05), Drug-drug interactions were found in 108 out of the 118 examined prescriptions, Total of 586 interactions in the admission list and 405 interactions in the discharge list.
